# Association of weight-adjusted-waist index with non-alcoholic fatty liver disease and liver fibrosis: a cross-sectional study based on NHANES

**DOI:** 10.1186/s40001-023-01205-4

**Published:** 2023-08-03

**Authors:** Qinggang Hu, Kexing Han, Jiapei Shen, Weijie Sun, Long Gao, Yufeng Gao

**Affiliations:** 1 Department of Infectious Diseases, The First Affiliated Hospital of Anhui Medical University, Hefei, Anhui 230022 China; 2 Department of Infectious Diseases, The First Affiliated Hospital of USTC, Division of Life Science and Medicine, University of Science and Technology of China, Hefei, Anhui 230001 China

**Keywords:** Weight-adjusted waist index, Non-alcoholic fatty liver disease, Liver fibrosis, Cross-sectional study, National health and nutrition examination survey

## Abstract

**Aim:**

The purpose of this study was to explore the association of weight-adjusted-waist index (WWI) with non-alcoholic fatty liver disease (NAFLD) and liver fibrosis.

**Methods:**

A cross-sectional study including 6587 participants was conducted in the National Health and Nutrition Examination Survey (NHANES). Multiple linear regression was used to validate the association of WWI with NAFLD and liver fibrosis, and smoothed curve fitting and threshold effect models were used to validate non-linear relationships. Subgroup analyses were used to verify the stability of the relationship between the independent and dependent variables in different populations.

**Results:**

There was a positive association of WWI with NAFLD and liver fibrosis. In the model adjusted for all covariates, the effect values of WWI with NAFLD and liver fibrosis were (OR = 3.44, 95% CI: 3.09–3.82) and (OR = 2.40, 95% CI: 2.05–2.79), respectively. This positive correlation became more significant as WWI increased when WWI was presented in quartiles (*P* for trend < 0.01). Smoothed curve fitting and threshold effects analysis suggested a non-linear correlation between WWI and NAFLD (LLR < 0.01), with the positive correlation between WWI and NAFLD becoming more significant when WWI was less than 11.44 [5.93 (95% CI: 5.04–6.98)]. However, there was a linear correlation between WWI and liver fibrosis (LLR = 0.291). When subgroup analyses were performed by indicators such as age, race and gender, we found that the positive association between WWI and the dependent variables (NAFLD and liver fibrosis) was more pronounced in white male participants aged < 40 years.

**Conclusions:**

Among adults in the United States, WWI was positively associated with the prevalence of NAFLD and liver fibrosis. Participants with a WWI less than 11.44 should be cautious about the possibility of an increased risk of NAFLD development due to a higher WWI. Meanwhile, white males younger than 40 years of age should be more cautious about the higher risk of NAFLD and liver fibrosis that might be associated with an increased WWI.

## Introduction

Non-alcoholic fatty liver disease (NAFLD) is currently the most common liver disease affecting approximately 2 billion people worldwide (with a prevalence of 25%) [1]. As research progresses, fatty liver disease is thought to be associated with metabolic dysfunction and all-cause mortality is much higher in patients with fatty liver disease than in the general population [2]. NAFLD is characterised by excessive lipid accumulation in the liver in the absence of alcohol abuse and may progress further to more advanced non-alcoholic steatohepatitis, fibrosis, cirrhosis and ultimately hepatocellular carcinoma [3]. Even so, the current gold standard for assessing the degree of steatosis and fibrosis in the liver is still liver biopsy. Because liver puncture is an invasive procedure that can be harmful to the human body, it needs to be used with caution in clinical practice [4]. Liver ultrasound transient elastography (LUTE) is a test that can assess the degree of liver fibrosis and fatty liver and is used in a large number of clinical applications due to its non-invasive [5].

The risk factors for NAFLD are complex, but previous studies have demonstrated that the prevalence of NAFLD was usually associated with obesity, but a significant proportion of patients are thin, which poses a challenge for screening for NAFLD [6].

Body mass index (BMI) is a traditional indicator for determining obesity, but its inability to distinguish between lean and fat body mass [7] has led to its accuracy being questioned in recent years [8, 9]. In recent years, it has been proposed that visceral fat more accurately reflects an unfavourable metabolic profile, which is often associated with abdominal obesity [10]. WWI is a simple anthropometric indicator based on BMI, calculated as WC (cm) divided by the square root of weight (kg) [11]. WWI mainly reflects the actual situation of central obesity due to the adjustment of body weight. While the central obese group is mainly the accumulation of visceral fat, the accumulation of visceral fat can cause more endocrine and metabolic diseases [12, 13]. A strong relationship between WWI and abdominal aortic calcification was demonstrated, with a near linear relationship between them [14]. It was also found that WWI was correlated with the development of hypertension, and the higher the WWI classification, the higher risk of hypertension [15]. In addition, WWI is strongly associated with all-cause mortality and cardiovascular mortality [16, 17]. However, there are still no studies correlating WWI with NAFLD and liver fibrosis.

In the present study, we aimed to assess the association of WWI with NAFLD and liver fibrosis in US adults. To achieve these objectives, we analysed data from the 2017–2020.03 cycle of the National Health and Nutrition Examination Survey (NHANES), where waist circumference and body weight were used to assess WWI and LUTE was used for liver steatosis and fibrosis.

## Materials

### Data sources

We conducted a cross-sectional study using information from the 2017–2020.03 NHANES database, which is a population-based omnibus survey. Questionnaires, physical examinations, and laboratory tests are used to obtain demographic, socioeconomic, dietary, and health-related information and to complete medical examinations that include anthropometric and laboratory assessments. The National Center for Health Statistics (NCHS) Ethics Review Committee approved the NHANES survey protocol, and all participants provided written informed consent. Because the NHANES database is open to the public, the ethical review of this study was exempt.

### Participants

**Fig. 1:**
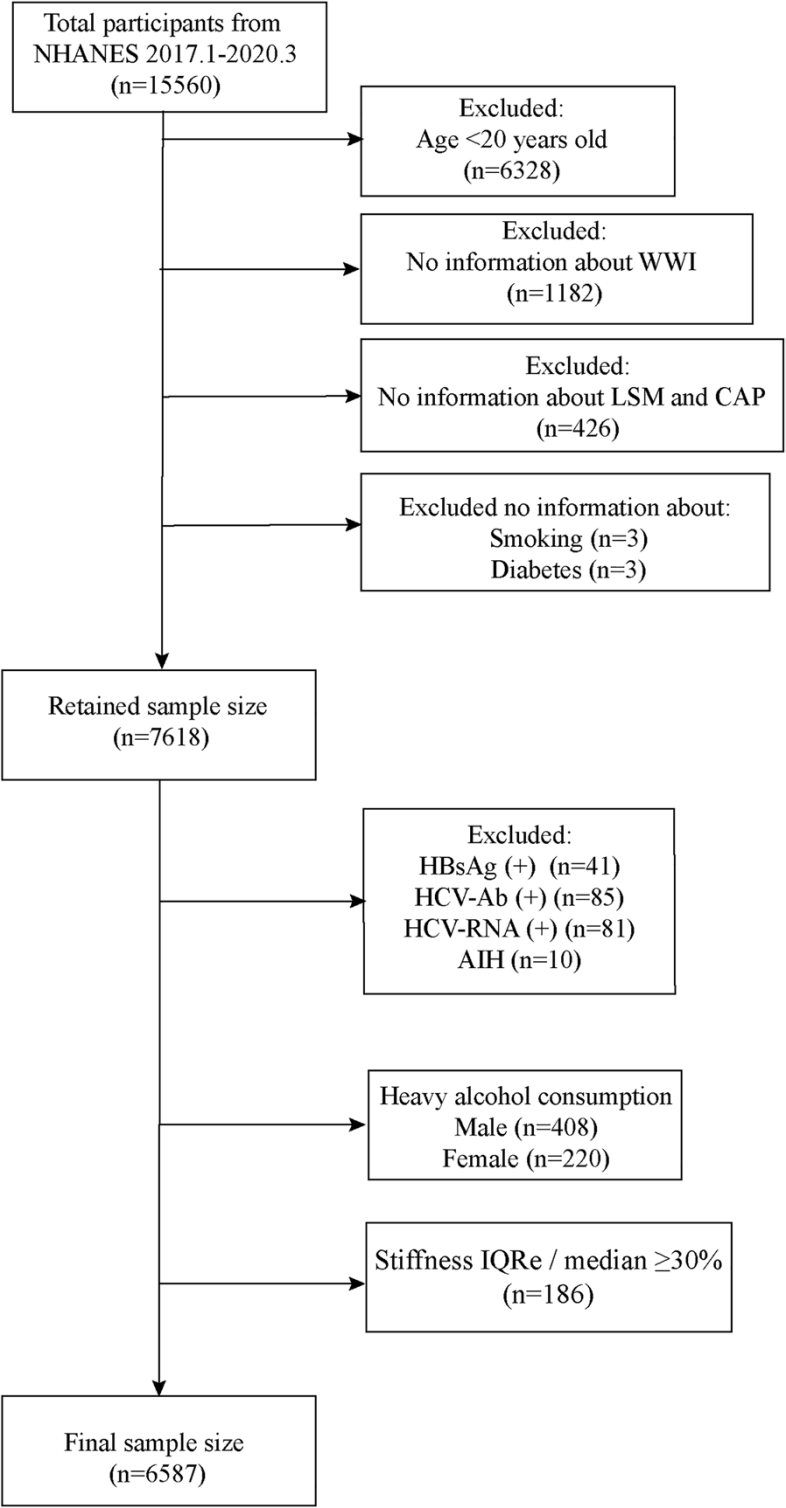
Flow chart for participants

A total of 15,560 participants in the NHANES database were included in the survey. We excluded participants who were younger than 20 years of age (*n* = 6328), had no WWI information (*n* = 1182) and had not completed the LUTE test (*n* = 427). A small number of participants with no life information were also excluded, including no smoking status (*n* = 3) and no information on diabetes (*n* = 3). Participants with a history of viral hepatitis were also excluded, including those who were positive for hepatitis B surface antigen (HBsAg) (*n* = 41), positive for hepatitis C antibody (HCV-Ab) (*n* = 85) and positive for hepatitis C virus RNA (HCV-RNA) (*n* = 81). Participants with autoimmune hepatitis (AIH) were also excluded (*n* = 10). Participants defined as consuming large amounts of alcohol [18] were excluded for males (alcohol intake > 30 g/d) (*n* = 508) and females (alcohol intake > 20 g/d) (*n* = 428). In accordance with NHANES guidelines, results were unreliable for liver stiffness measures (LSM) at an interquartile range (IQRe)/median of > 30% and we also excluded this group of participants (*n* = 186). Ultimately, the remaining 6587 participants were included in the study. The specific flow chart is shown in Fig. 1.

### Assessment of NAFLD and liver fibrosis

The main objective of LUTE is to provide an objective measurement of two important manifestations of liver disease: liver fibrosis (liver scarring) and liver steatosis (liver fat). All measurements were performed on the FibroScan^®^ machine and one of the physical parameters, which we could call the Controlled Attenuation Parameter (CAP), was measured to reflect mainly the degree of hepatic steatosis. A median value of ≥ 274 dB/m for CAP was considered to be a marker of steatosis according to a study conducted by Eddowes et al. [19]. Liver fibrosis was judged based on liver stiffness measurements (LSM) and according to the latest guidelines of the European Association for the Study of the Liver [20], a median LSM of ≥ 8.0 kPa was considered to be the presence of liver fibrosis (≥ F2) [21]. All participants taking the test were aged 12 years and older. Participants who could not lie on the examination table, were pregnant at the time of the test (or were unsure if they were pregnant) or could not obtain urine for a pregnancy test, had an electronic medical device implanted, or were wearing a bandage or had an injury to the right rib cage of the abdomen (to be measured) needed to be excluded.

### Calculation of weight-adjusted waist index (WWI)

The indicators used to calculate WWI were obtained from body measurement information in the physical examination module. Body measurement data were collected by trained health technicians in mobile examination centres (MECs).WWI was calculated by dividing waist circumference in centimetres by the square root of body weight in kilograms [22]. Body measurement information for the NHANES 2017–2020.03 cycle was reviewed and Measurements remain consistent over this period.

### Covariates

With the previous study, we adjusted the covariates added to the model (19–22). First, the covariates to be adjusted for in the demographic information include age, gender (male, female), race (White, Black and Other races), education (less than high school and high school, greater than high school), and family income to poverty (PIR). Physical examination included height (cm). Clinical data included C-reactive protein (CRP), serum ferritin. The questionnaire included smoking, hypertension, diabetes, blood transfusion, and sedentary. Smoking was defined as smoking at least 100 cigarettes in a lifetime and was categorised into three states (ever, now, and never) based on whether or not they currently smoked. Dietary information includes energy intake, sugar intake, fat intake and water intake. The data were obtained in the form of a questionnaire calculated as the average of the sum of the values of the ingested substances answered on day 1 and day 2. All covariates are presented in Table 1.

**Table 1:** Characteristics of the participants

Characteristics	Group 1	Group 2	P value
Sample size	3293	3294	
Age(years)	43.49 ± 15.89	57.36 ± 16.04	< 0.001
Stratified by age (years), *n* (%)			< 0.001
< 40	1494 (45.37)	553 (16.79)	
40–59	1163 (35.32)	1045 (31.72)	
> 60	636 (19.31)	1696 (51.49)	
Gender, *n* (%)			< 0.001
Male	1869 (56.76)	1307 (39.68)	
Female	1424 (43.24)	1987 (60.32)	
Race, *n* (%)			< 0.001
White	975 (29.61)	1224 (37.16)	
Black	996 (30.25)	732 (22.22)	
Other race	1322 (40.15)	1338 (40.62)	
Height (cm)	169.52 ± 9.60	163.29 ± 9.46	< 0.001
Education, *n* (%)			< 0.001
Less than high school, high school	1223 (37.14)	1602 (48.63)	
More than high school	2067 (62.77)	1687 (51.21)	
Unclear	3 (0.09)	5 (0.15)	
PIR, *n* (%)			< 0.001
< 1.35	742 (22.53)	890 (27.02)	
1.35–3.45	1017 (30.88)	1162 (35.28)	
> 3.45	1087 (33.01)	798 (24.23)	
Unclear	447 (13.57)	444 (13.48)	
Smoking, *n* (%)			< 0.001
Ever	588 (17.86)	916 (27.81)	
Now	617 (18.74)	473 (14.36)	
Never	2088 (63.41)	1905 (57.83)	
Hypertension, *n* (%)			< 0.001
Yes	819 (24.87)	1635 (49.64)	
No	2471 (75.04)	1654 (50.21)	
Unclear	3 (0.09)	5 (0.15)	
Diabetes, *n* (%)			< 0.001
Yes	220 (6.68)	754 (22.89)	
No	2998 (91.04)	2406 (73.04)	
Unclear	75 (2.28)	134 (4.07)	
Blood transfusion, *n* (%)			< 0.001
Yes	266 (8.08)	431 (13.08)	
No	3007 (91.31)	2801 (85.03)	
Unclear	20 (0.61)	62 (1.88)	
Total daily energy intake (kcal), *n* (%)			< 0.001
< 1861.5	1138 (34.56)	1458 (44.26)	
≥ 1861.5	1435 (43.58)	1162 (35.28)	
Unclear	720 (21.86)	674 (20.46)	
Total daily sugar intake (gm), *n* (%)			< 0.001
< 88.44	1210 (36.74)	1386 (42.08)	
≥ 88.44	1363 (41.39)	1234 (37.46)	
Unclear	720 (21.86)	674 (20.46)	
Total daily fat intake (gm), *n* (%)			< 0.001
< 76.35	1179 (35.80)	1417 (43.02)	
≥ 76.35	1394 (42.33)	1203 (36.52)	
Unclear	720 (21.86)	674 (20.46)	
Total daily moisture intake (gm), *n* (%)			< 0.001
< 2454.88	1215 (36.90)	1381 (41.92)	
≥ 2454.88	1358 (41.24)	1239 (37.61)	
Unclear	720 (21.86)	674 (20.46)	
CRP (mg/l)	2.82 ± 5.04	5.18 ± 10.09	< 0.001
Ferritin (ng/ml)	153.57 ± 162.07	157.61 ± 179.72	0.905
Activity intensity (min)	324.85 ± 200.51	329.10 ± 200.72	0.245
Stratified by CAP (dB/m), *n* (%)			< 0.001
< 274	2343 (71.15)	1370 (41.59)	
≥ 274	950 (28.85)	1924 (58.41)	
Stratified by LSM (Kpa), *n* (%)			< 0.001
< 8.0	3141 (95.38)	2796 (84.88)	
≥ 8.0	152 (4.62)	498 (15.12)	

Mean ± SD for continuous variables: P value was calculated by weighted linear regression model

% For Categorical variables: *p* value as calculated by weighted Chi-square test

### Statistical analysis

Data collation and statistical analysis were done via R (4.1.2) and Empower Stats. Due to the 2019 coronavirus disease (COVID-19) pandemic, the NHANES project suspended field operations in March 2020. As a result, data collected from 2019 to March 2020 were combined with data from the NHANES 2017–2018 cycle to form a nationally representative sample. The NHANES working group applied a special weighting process to the pre-epidemic data files for March 2017–2020. In accordance with NHANES guidelines, NHANES check sample weights were applied to the analysis of the LUTE data. Therefore, the special examination sample weights (Variable Name: WTMECPRP) for the 2017–2020.03 cycle were used in this study. Means ± standard errors were used to represent continuous variables, and values and percentages were used to represent categorical variables. Multiple linear regression analysis was used to examine the relationship between the independent and dependent variables. A total of three models were generated based on the adjustment of covariates. Model 1: No adjustment for covariates. Model 2: Adjusted for age, race and gender. Model 3: All covariates in Table 1 are adjusted. Subgroup analysis was then used to find a more sensitive cohort. Subgroup analysis was used to assess whether the correlations between the independent and dependent variables were stable across cohorts and to find sensitive populations. A smoothed curve fitting analysis was used to verify whether there was a non-linear relationship between the independent and dependent variables and to verify this using a threshold effect model. For the threshold effects analysis, a log-likelihood ratio (LLR) of less than 0.05 was used as the criterion for the presence of a non-linear relationship.

## Results

### Participant characteristics

Eventually, a total of 6587 participants were collected in this study. We grouped the participants based on the median WWI (11.11) for Group 1 (< 11.11) and Group 2 (> 11.11). The results of the comparison between groups suggested that participants with a WWI > 11.11 had higher rates of NAFLD and liver fibrosis (*p* < 0.05). The results of participant characteristics are presented in Table 1.

### The association of WWI with NAFLD and liver fibrosis

Based on the results in Table 2, there was a positive association of WWI with NAFLD and liver fibrosis in all models. Across all participants, the positive effect between WWI and NAFLD was (OR = 3.44, 95% CI: 3.09–3.82), and the positive effect with liver fibrosis was (OR = 2.40, 95% CI: 2.05–2.79). When we grouped WWI according to quartiles, we found that this correlation persisted. Meanwhile, we found that the association of WWI with NAFLD and liver fibrosis would increase with increasing WWI (*P* for trend < 0.0001). In the fourth quartile group of WWI (11.698–14.137), the positive effect between WWI and NAFLD was (OR = 14.65, 95% CI: 11.52–18.64) as well as the positive effect with liver fibrosis (OR = 5.61, 95% CI: 3.86–8.15).

**Table 2:** Association of WWI with NAFLD and liver fibrosis

Exposure	Model 1, β (95% CI)	Model 2, β (95% CI)	Model 3, β (95% CI)
NAFLD			
WWI	2.53 (2.37, 2.71)	3.28 (3.01, 3.56)	3.44 (3.09, 3.82)
Quintiles of WWI			
Q1(8.443–10.534)	Reference	Reference	Reference
Q2(10.535–11.109)	3.72 (3.16, 4.38)	4.23 (3.56, 5.02) < 0.0001	4.29 (3.51, 5.24)
Q3(11.110–11.697)	6.16 (5.23, 7.25)	8.06 (6.73, 9.66) < 0.0001	8.61 (6.95, 10.67)
Q4(11.698–14.137)	8.75 (7.42, 10.32)	13.99 (11.50, 17.03)	14.65 (11.52, 18.64)
*P* for trend	< 0.0001	< 0.0001	< 0.0001
Liver fibrosis			
WWI	2.13 (1.93, 2.36)	2.48 (2.19, 2.80)	2.40 (2.05, 2.79)
Quintiles of WWI			
Q1(8.443–10.534)	Reference	Reference	Reference
Q2(10.535–11.109)	1.36 (0.98, 1.89)	1.42 (1.01, 1.98)	1.36 (0.93, 1.98)
Q3(11.110–11.697)	3.29 (2.46, 4.39)	3.62 (2.67, 4.93)	2.94 (2.06, 4.20)
Q4(11.698–14.137)	5.46 (4.14, 7.21)	6.85 (5.00, 9.37)	5.61 (3.86, 8.15)
*P* for trend	< 0.0001	< 0.0001	< 0.0001

### Smooth curve fitting and threshold effect analysis

**Fig. 2:**
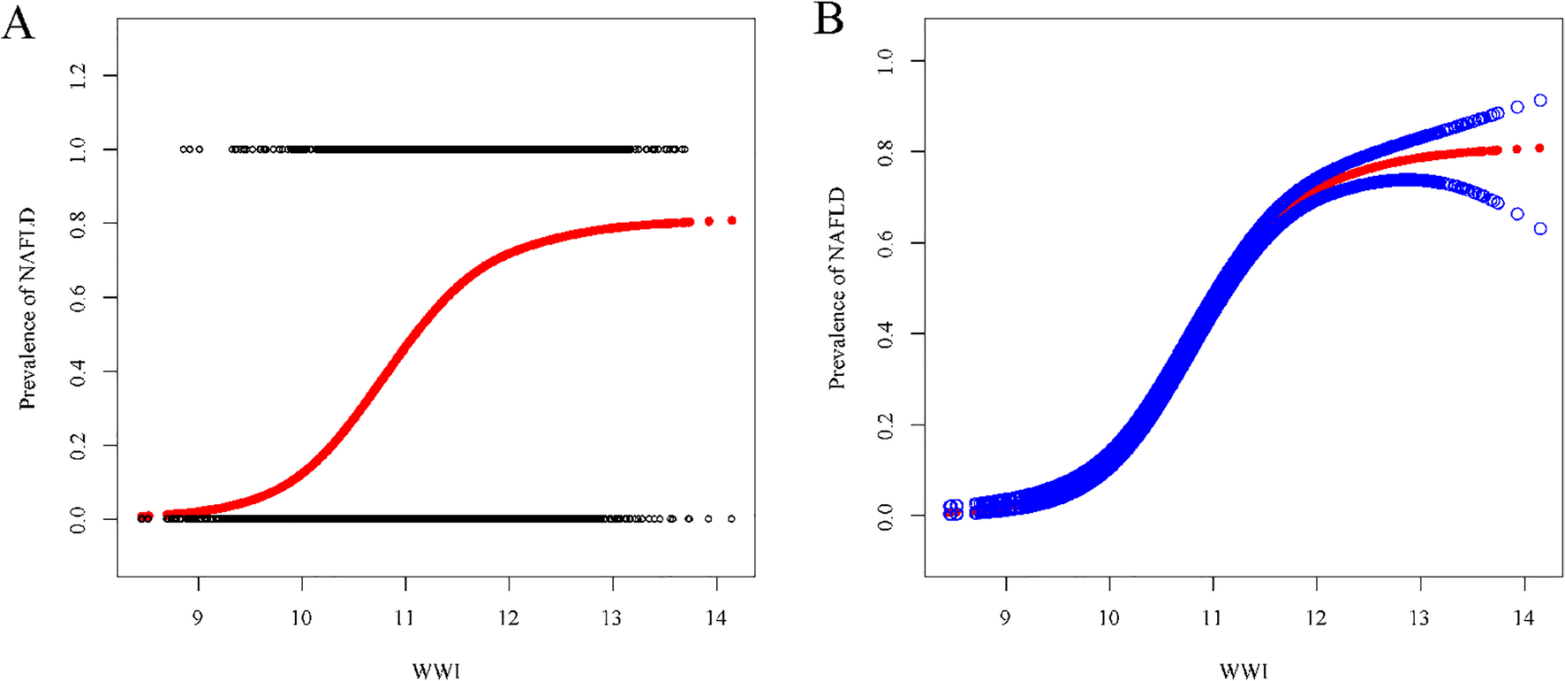
The association between WWI and NAFLD. **a** Each black point represents a sample. **b** Solid red line represents the smooth curve fit between variables. Blue bands represent the 95% of confidence interval from the fit. All the covariates in Table 1 are adjusted

**Fig. 3:**
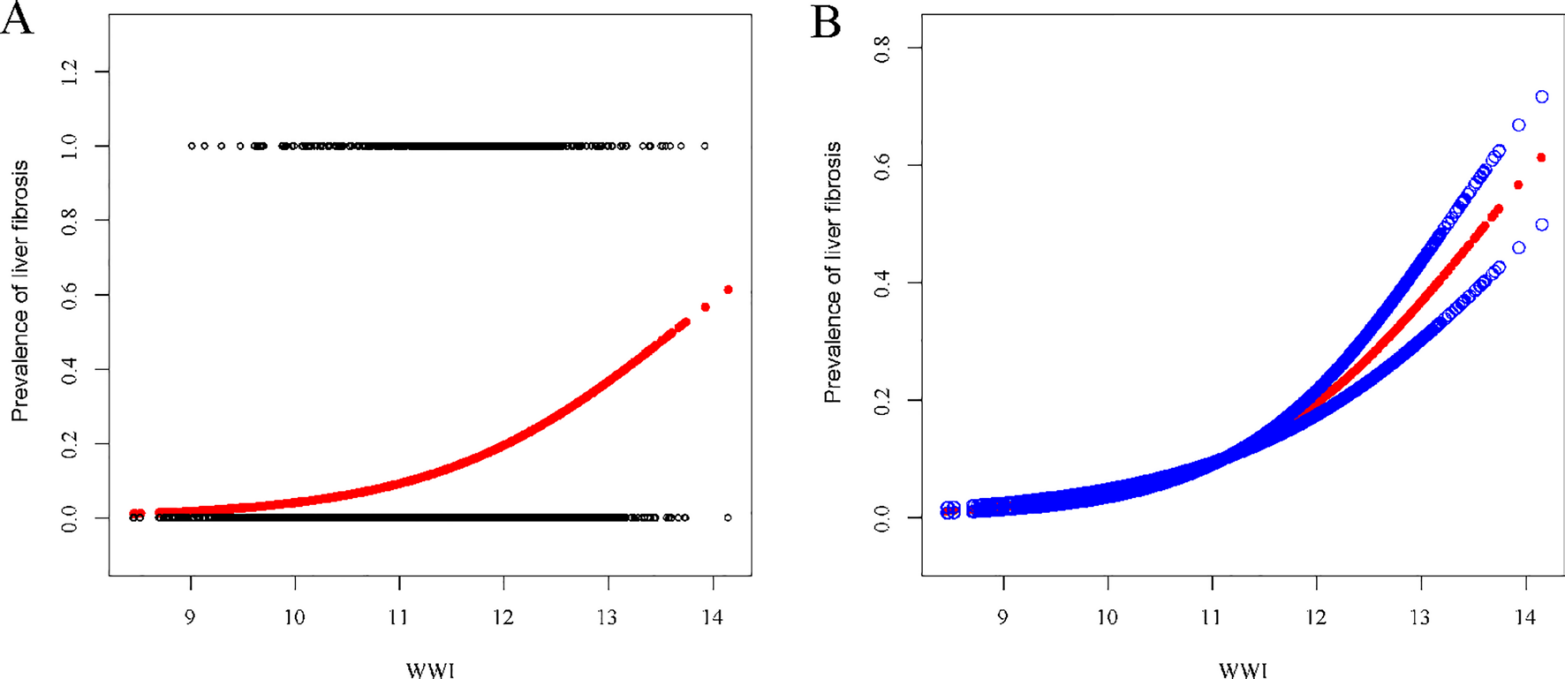
The association between WWI and liver fibrosis. **a** Each black point represents a sample. **b** Solid red line represents the smooth curve fit between variables. Blue bands represent the 95% of confidence interval from the fit. All the covariates in Table 1 are adjusted

Through smoothed curve fitting analysis, we explored whether the positive association of WWI with NAFLD and liver fibrosis was non-linear and verified with a threshold effect. Based on Fig. 2, we found that there was not a perfectly linear association between WWI and either of the two dependent variables. The results of the threshold effect model suggested a non-linear correlation between WWI and NAFLD (LLR < 0.001). The positive relationship between WWI and NAFLD was more significant at WWI less than 11.44, with an effect value of (OR = 5.93, 95% CI: 5.04–6.98). However, a linear relationship was found between WWI and liver fibrosis (LLR = 0.291). The results of the smoothed curve fitting and threshold effect analysis were displayed in Fig. 3 and Table 3.

**Table 3:** Threshold effect analysis for association of WWI with NAFLD and liver fibrosis

Outcomes	NAFLD	Liver fibrosis
Model 1, β (95% CI)		
Linear effect model	3.44 (3.09, 3.82)	2.40 (2.05, 2.79)
Model 2, β (95% CI)		
Inflection point (K)	11.44	10.56
< K	5.93 (5.04, 6.98)	1.80 (1.06, 3.07)
> K	1.55 (1.28, 1.88)	2.51 (2.10, 2.99)
LLR	< 0.001	0.291

### Results of subgroup analysis

In the subsequent subgroup analyses, we focused on gender, age and races. We first analysed gender. The positive association of WWI with NAFLD and liver fibrosis was stable across all genders, but the correlation was more pronounced in male participants than in female participants. We then divided the participants into three groups according to their age. On the basis of the results, it was suggested that the positive association of WWI with NAFLD and liver fibrosis remained stable across all age groups. However, this positive correlation was attenuated with increasing age. Finally, we found that the positive correlation between WWI and NAFLD and liver fibrosis was more significant in white participants. The results of subgroup analyses for the association of WWI with NAFLD and liver fibrosis are shown in Tables 4 and 5, respectively.

**Table 4:** Subgroup analysis of association between WWI and NAFLD

Characteristics	Model 1, β (95% CI)	Model 2, β (95% CI)	Model 3, β (95% CI)
Stratified by gender			
Male	3.52 (3.14, 3.94)	4.89 (4.23, 5.65)	5.92 (4.89, 7.17)
Female	2.53 (2.30, 2.79)	2.55 (2.29, 2.82)	2.54 (2.23, 2.88)
Stratified by age(years)			
< 40	3.15 (2.74, 3.61)	4.56 (3.88, 5.37)	4.30 (3.52, 5.25)
40–59	2.97 (2.59, 3.41)	3.53 (3.05, 4.10)	3.40 (2.82, 4.10)
≥ 60	2.07 (1.83, 2.34)	2.21 (1.94, 2.52)	2.69 (2.26, 3.20)
Stratified by race			
White	2.56 (2.28, 2.88)	3.48 (3.01, 4.03)	3.77 (3.13, 4.54)
Black	2.54 (2.22, 2.89)	2.99 (2.56, 3.49)	3.22 (2.67, 3.89)
Other race	2.45 (2.19, 2.74)	3.25 (2.83, 3.74)	3.47 (2.89, 4.17)

**Table 5:** Subgroup analysis of association between WWI and liver fibrosis

Characteristics	Model 1, β (95% CI)	Model 2, β (95% CI)	Model 3, β (95% CI)
Stratified by gender			
Male	2.43 (2.08, 2.84)	2.65 (2.22, 3.17)	2.55 (2.02, 3.21)
Female	2.44 (2.09, 2.85)	2.34 (1.98, 2.76)	2.37 (1.92, 2.92)
Stratified by age (years)			
< 40	2.68 (2.13, 3.37)	3.49 (2.69, 4.52)	3.75 (2.65, 5.30)
40–59	2.49 (2.06, 3.00)	2.91 (2.37, 3.57)	2.57 (1.97, 3.34)
≥ 60	1.51 (1.28, 1.79)	1.78 (1.48, 2.14)	1.74 (1.38, 2.21)
Stratified by race			
White	2.47 (2.06, 2.94)	2.92 (2.35, 3.63)	3.28 (2.44, 4.41)
Black	1.87 (1.56, 2.24)	1.89 (1.52, 2.35)	1.87 (1.43, 2.45)
Other race	2.08 (1.76, 2.46)	2.67 (2.18, 3.27)	2.44 (1.89, 3.16)

## Discussion

NAFLD is now prevalent worldwide, with an average global prevalence of 20–30%. Timely assessment of the risk of developing NAFLD is crucial for patients, and all efforts lie in stopping the disease from progressing to the advanced fibrosis stage, due to the fact that advanced fibrosis increases the risk of hepatocellular carcinoma and other complications of cirrhosis [23]. Given the tendency of patients to avoid liver biopsy, the most accurate non-invasive method that can be accepted is currently based on liver elastography [24]. Several studies have found a strong association between BMI and the development of NAFLD and liver fibrosis [25, 26]. However, no articles have reported the association of WWI with NAFLD and liver fibrosis. In our study, we found that WWI was a strong factor positively influencing the development of NAFLD and liver fibrosis. This positive correlation persisted even when we grouped WWI according to quartiles. Moreover, this positive correlation increased with increasing WWI.

As mentioned earlier, the accuracy of BMI as a traditional indicator for assessing obesity has been questioned. In order to better reflect the true reality of obesity, a new obesity index was proposed and named the Weight Adjusted Waist Index (WWI) [11]. As weight is adjusted in the calculation of the WWI, this index mainly reflects weight-independent central obesity. The WWI has been shown to have better accuracy compared to BMI [15, 17, 27]. Based on the BMI formula, changes in body weight were often used to reflect fat accumulation and obesity alone in the adult population due to the stability of height. However, in recent years this notion has been questioned by researchers [28, 29]. Even more notably, when the concept of the muscle–fat–liver axis was introduced, researchers recognised that weight loss was also likely to be caused by a loss of muscle mass and that this process was often accompanied by the accumulation of visceral fat, which may reflect a more accurate level of obesity [30]. It is undeniable that NAFLD [31] is a disease closely associated with inflammation, and chronic inflammatory stimulation of the liver in particular is key to the progression of NAFLD into liver fibrosis [32]. Furthermore, obesity has been demonstrated as a low-grade inflammatory state of the organism [33, 34]. Therefore, it would be accepted that inflammation may be a key factor mediating the link between obesity and NAFLD as well as liver fibrosis. More studies have shown that central obesity was more closely associated with inflammation and metabolic disorders [11, 35]. This was therefore an advantage that our study had.

In addition, we confirmed sensitive populations with positive correlations between WWI and the dependent variables, either with NAFLD or liver fibrosis as the dependent variable. The first population characteristic that was validated was male. It is known from previous epidemiological studies that male have higher rates of NAFLD and liver fibrosis compared to female when holding other covariates constant [36, 37]. In addition, when BMI was used as a criterion for determining obesity, previous studies reported that obesity was more prevalent in women worldwide [38, 39]. However, women are predominantly peripherally obese, as they are characterised by fat deposits in the buttocks, thighs and limbs and subcutaneous tissue, and have a pear-shaped body, in contrast to men who are predominantly obese with increased visceral fat (central obesity) [9]. In fact, visceral fat is more active than subcutaneous fat, and there is a closer link between it and metabolic inflammation [40]. In addition to this, recent studies have shown that the increased fat may be more significant in the incidence of liver fibrosis in women in the abdominal obesity pattern [41]. Typically, older people have increased fat deposits in tissues such as the heart, liver and skeletal muscle [42]. However, in one study, Amdanee N performed dual-energy X-ray absorptiometry (DXA) measurements on 102 men aged 31–83 years to assess the effect of age on fat distribution (both subcutaneous and visceral fat). The results showed that the rate of subcutaneous and visceral fat accumulation was negatively correlated with age [43]. We agree that older age groups have higher rates of obesity, NAFLD and liver fibrosis, and in Table 1 our results also suggested a higher proportion of participants with a WWI > 11.11 with increasing age. However, younger groups appear to be more sensitive to increases in WWI [43]. In addition, in the elderly, there is essentially a linear relationship between WWI and LSM. This is due to the fact that liver fibrosis is already at a higher level in the elderly and therefore its changes are flatter, as demonstrated by Klisic's study [44]. Kim D reported racial differences in NAFLD and liver fibrosis in the US population. Between 2013 and 2016, the prevalence of NAFLD and liver fibrosis steadily increased in non-Hispanic whites, but the late prevalence was flat in non-Hispanic blacks [45]. Epidemiological studies suggested that adult obesity rates in the United States have steadily increased over the past decade and have stabilised at a prevalence of approximately 35%, compared with 43% and 48% for Hispanics and non-Hispanic blacks, respectively [46]. However, according to Staiano et al. they found that fat distribution characteristics differed by race, with white people being more likely to have fat distribution in the visceral organs than black people [47].

This study has important clinical value. It was the first study to use the new obesity index (WWI) to explore the association of obesity with NAFLD and liver fibrosis, and our study sample size was adequate and representative. Through subgroup analysis, we further understand the characteristics of different ages, genders and ethnicities in this correlation, which could guide us in giving different recommendations to different populations in clinical practice. However, there were still several limitations to our study. Firstly, the age of the participants in this study was above 20 years old, which leads to the possibility that our findings may not apply to participants under 20 years old. Therefore, further exploration of those under 20 years of age is needed. Secondly, this study included a number of covariates from the questionnaire which may be influenced by individual subjective factors. Third, our study was a cross-sectional study, which cannot explain causal relationships. In addition, there are numerous potential influences on obesity, NAFLD and liver fibrosis, and even though we included as many covariates as possible in our study to adjust for them in the model, there was still no guarantee that there were potential confounders leading to bias in the results. Therefore, more prospective studies are needed. Finally, the diagnosis of NAFLD and liver fibrosis in this study was not confirmed by liver biopsy, so the current conclusions need to be confirmed by follow-up studies.

## Conclusion

In this study, WWI was positively correlated with both NAFLD and liver fibrosis in US adults, with NAFLD and liver fibrosis after LUTE discrimination as the dependent variables. The positive relationship between WWI and NAFLD was non-linear, and this positive correlation was more pronounced for WWI less than 11.44. The positive association of WWI with either NAFLD or liver fibrosis was more pronounced in white participants younger than 40 years of age.

## Data Availability

Article/Additional files include the original contributions presented in the study. Please contact the corresponding authors for further information.
